# The use of recombinant human growth hormone in patients with Mucopolysaccharidoses and growth hormone deficiency: a case series

**DOI:** 10.1186/s13052-019-0691-1

**Published:** 2019-08-01

**Authors:** A. Cattoni, S. Motta, N. Masera, S. Gasperini, A. Rovelli, R. Parini

**Affiliations:** 1 Paediatric Department, Azienda Ospedaliera San Gerardo – Fondazione Monza e Brianza per il Bambino e la sua Mamma, Via Pergolesi 33, 20900 Monza, MB Italy; 2 TIGET Institute, IRCCS San Raffaele Hospital, Via Olgettina 60, 20132 Segrate, MI Italy

**Keywords:** Mucopolysaccharidoses, Pituitary dwarfism, Human growth hormone, Failure to thrive

## Abstract

**Background:**

The treatment with recombinant human growth hormone in patients affected by Mucopolysaccharidoses (MPS) is considered whenever a concurrent diagnosis of growth hormone deficiency is demonstrated. The short- and long-term effects of recombinant human growth hormone in this selected cohort is still debated, given the natural progression of disease-related skeletal malformations and the paucity of treated patients reported in literature. The presented case series provides detailed information about the response to recombinant growth hormone in MPS patients diagnosed with growth hormone deficiency.

**Cases presentation:**

The growth patterns of 4 MPS female patients (current age: 11.7–14.3 years) treated with recombinant human growth hormone due to growth hormone deficiency have been retrospectively analyzed. Two patients, diagnosed with MPS IH, had undergone haematopoietic stem cell transplantation at an early age; the remaining two patients were affected by MPS IV and VI and were treated with enzyme replacement therapy.

4/4 patients presented with a progressive growth deceleration before the diagnosis of growth hormone deficiency was confirmed. This trend was initially reverted by a remarkable increase in height velocity after the start of recombinant growth hormone. We recorded an average increase in height velocity z-score of + 4.23 ± 2.9 and + 4.55 ± 0.96 respectively after 6 and 12 months of treatment. After the first 12–24 months, growth showed a deceleration in all the patients. While in a girl with MPS IH recombinant human growth hormone was discontinued due to a lack in clinical efficacy, 3/4 patients grew at a stable pace, tracking the height centile achieved after the cited initial increase in height velocity.

Furthermore, mineral bone density assessed via bone densitometry, showed a remarkable increase in the two patients who were tested before and after starting treatment.

**Conclusions:**

Recombinant human growth hormone appears to have effectively reverted the growth deceleration experienced by MPS patients diagnosed with growth hormone deficiency, at least during the first 12–24 months of treatment.

## Background

Mucopolysaccharidoses (MPS) are a heterogeneous group of inherited lysosomal storage disorders arising from the defective activity of lysosomal enzymes involved in the process of degradation of mucopolysaccharides (or glycosaminoglycans (GAG)). The consequent accumulation of different GAG leads to a clinically progressive disease with multi-systemic complications.

Most patients diagnosed with MPS experience a variable degree of faltering growth and short stature [1]. The pathogenesis of short stature in MPS is not completely understood, although the progressive accumulation in cartilage and bones is identified as the “primum movens” of bones abnormalities in these patients. Simonaro and colleagues reported that GAG storage induces a complex cascade of secondary and tertiary effects, leading to inflammation, apoptosis of cartilage cells, and hyperplasia of synovial membranes, resulting in poorly organized and metabolically abnormal connective tissue matrix [2]. This disruption of cell functions is considered responsible of the diffuse skeletal deformities (i.e. kyphosis, scoliosis, *genu valgum*) and reduced growth potential in MPS. Metabolic and endocrine (growth hormone deficiency, precocious puberty, hypothyroidism) abnormalities have also been reported in a number of cases contributing to reduced growth [3–5].

MPS patients treated with Enzyme Replacement Therapy (ERT) plus Haematopoietic Stem Cell Transplantation (HSCT) if affected by MPS IH (Hurler syndrome) or with ERT alone when affected by MPS I H/S (Hurler/Scheie syndrome), MPS IS (Scheie syndrome), MPS II (Hunter syndrome), IV (Morquio syndrome) and VI (Maroteaux-Lamy syndrome), experience a remarkable improvement in their abdominal, respiratory and cardiovascular features [6, 7]; however, the effects of HSCT and ERT on musculoskeletal system and growth are frequently not satisfying [6, 8]. Harmatz and colleagues have reported an early mild increase in height Standard Deviation Score (SDS) in MPS VI patients on ERT [9]; Jones and colleagues have described a similar response to ERT in subjects diagnosed with MPS II [10]. However, the reported results were age-dependent and the overall effect of ERT on height gain was limited.

Given the described detrimental effects of radiation on final stature, the assessment of growth after HSCT may result in misleading conclusions in patients for whom total body irradiation (TBI) was part of conditioning schedules before HSCT [11].

Only a few manuscripts have reported the growth patterns and eventual side effects after starting recombinant human growth hormone (rhGH) in GH-deficient patients with mucopolysaccharidoses: Polgreen and colleagues have provided figures about 6 MPS IH children treated with rhGH, among whom only 2 were GH-deficient, while 4 were treated despite normal GH response to GH stimulation tests (GH peak > 10 microg/L): no remarkable differences in height velocity were noticed between treated and untreated patients, although the sub-group of GH-deficient patients showed a better response to treatment when compared to non-GH deficient subjects [12]. Rogers and colleagues have described a single patient diagnosed with MPS I H/S, who presented a significantly improved linear growth after starting her on recombinant growth hormone [13]. One patient with growth hormone deficiency (GHD) and MPS II has been reported [3], while there are no available published data about GH-deficient subjects with MPS III, IV, VI and VII.

In order to describe both the growth patterns and the orthopaedic outcomes of MPS patients treated with rhGH for GHD, we hereby report about 4 GH-deficient subjects with an underlying diagnosis of MPS IH (2 subjects), MPS IV and MPS VI.

## Patients and methods

Among 64 MPS patients followed at the Metabolic Unit in Monza, 4 MPS female patients (two patients with MPS IH, one with MPS IV and one with MPS VI; current age: 11.7–14.3 years), have been found to be GH-deficient and were therefore started on rhGH therapy.

Clinical, radiological and auxological data were retrospectively collected from patients’ clinical reports and paper growth charts. Standing heights, weights and Tanner stages were assessed by a single experienced paediatric endocrinologist (NM). Standing heights were measured by wall-mounted stadiometer (without shoes) to the nearest 0.1 cm; weight was measured to the nearest 0.1 kg.

Growth centiles and height standard deviation scores (SDS, z-scores) were calculated according to the CDC 2002 growth charts with Lily’s Growth4® software. For patients with MPS IV and MPS VI, we have also plotted patient’s heights on the disease-specific growth curves for Morquio and Maroteaux-Lamy patients [14, 15]. For the patient with MPS VI, height SDS were calculated also according to the disease-specific growth charts using the LMS method [16].

GH *status* assessment was performed via arginine and either dexamethasone or insulin tolerance test (ITT); GHD was defined as an insufficient response (GH peak < 10 before 2010 and < 8 microg/L after 2010) at two sequential tests, performed in two different days. GH levels were assessed with the DiaSorin Liaison® XL assay.

Arginine was dispensed as a 30 min intravenous infusion (dose: 0.5 g/kg, maximum 30 g) and blood samples for GH and blood glucose were withdrawn at the following time points: − 15 mins, time 0, + 30 mins, + 45 mins, + 60 mins, + 75 mins, + 90 mins, + 105 mins and + 120 mins.

Dexamethasone was administered intravenously at the dose of 2 mg/m^2^; blood samples for GH assessment were withdrawn at the following time points: + 60′, + 90′, + 120′, + 135′, + 150′, + 165′, + 180′, + 195′, + 210′,+ 225′, + 240′.

Finally, during ITT, regular insulin is administered intravenously at a variable dose, according to the baseline blood glucose levels (doses between 0.05 and 0.1 IU/kg). Insulin-induced hypoglycaemic stimulus was considered adequate when at least one of the following conditions occurred: a drop in blood glucose levels to 45 mg/dL or less; a decrease in blood glucose levels by at least 50% from basal values; the child became symptomatic. Blood samples for GH and blood glucose levels were withdrawn at the following time points: − 20 mins, time 0, + 20 mins, + 30 mins, + 45 mins, + 60 mins, + 90 mins and + 120 mins.

According to indications provided by the Paediatric Endocrine Society [17], sex steroid priming prior to provocative GH testing was not performed because our patients were younger than 10 years.

Given the variability of normal values with gender, age and laboratory assay used, we recorded IGF-1 levels as standard deviation scores (IGF-1 SDS) according to age- and sex-adjusted reference ranges for each kit (lower cut-off: − 2 SDS, upper cut-off: + 2 SDS).

The doses of rhGH administered were the same as those employed for non-MPS subjects diagnosed with GHD, i.e. 0.025–0.035 mg/kg/die or, alternatively, 5–7 mg/m^2^/week.

Average treatment duration for the patients described was 3.3 ± 1.9 years (range 3.0 to 6.2).

Clinical and auxological assessments in patients treated with rhGH were systematically performed every 6 months (± 1 month). Height velocity was estimated over time intervals not shorter than 6 months; height velocity SDS were calculated according to the WHO 2006 charts.

Informed consent was obtained from patient’s guardians for publication of the case series and accompanying images.

## Case series

### Hurler syndrome (MPS 1H)

Two MPS 1H patients were diagnosed with GHD and treated with rhGH in our Centre. They both received treatment with ERT in the first months of life followed by early HSCT. Both these patients received a busulphan−/cyclophosphamide-based myeloablative conditioning regimen before HSCT.

Patient 1 was started on ERT at the age of 15 months and underwent HSCT at 20 months of life. Due to a precocious loss of engraftment, a second HCST was successfully performed at the age of 2.8 years and adequate levels of circulating enzyme during the subsequent 11 years follow-up confirmed the stable persistence of engraftment.

At the age of 6.7 years, the combination of progressive growth deceleration at clinical review (height velocity: 1.8 cm/year, − 5.14 SDS), IGF-1 levels below − 2 SDS and a remarkably delayed bone age (4.5 years compared to a chronological age of 6.7 years), prompted the clinicians to perform arginine and dexamethasone stimulation tests, which confirmed the diagnosis of GH deficiency (GH peaks achieved: 7.9 and 4.4 microg/L, respectively).

MRI showed a normal appearance of the hypothalamic-pituitary area.

She was therefore started on a standard dose of rhGH (0.025 mg/kg/die) and it was followed by a normalization of IGF-1 levels at subsequent biochemical follow-up, with IGF-1 SDS persistently between + 1.5 and + 1.9 SDS.

**Fig. 1:**
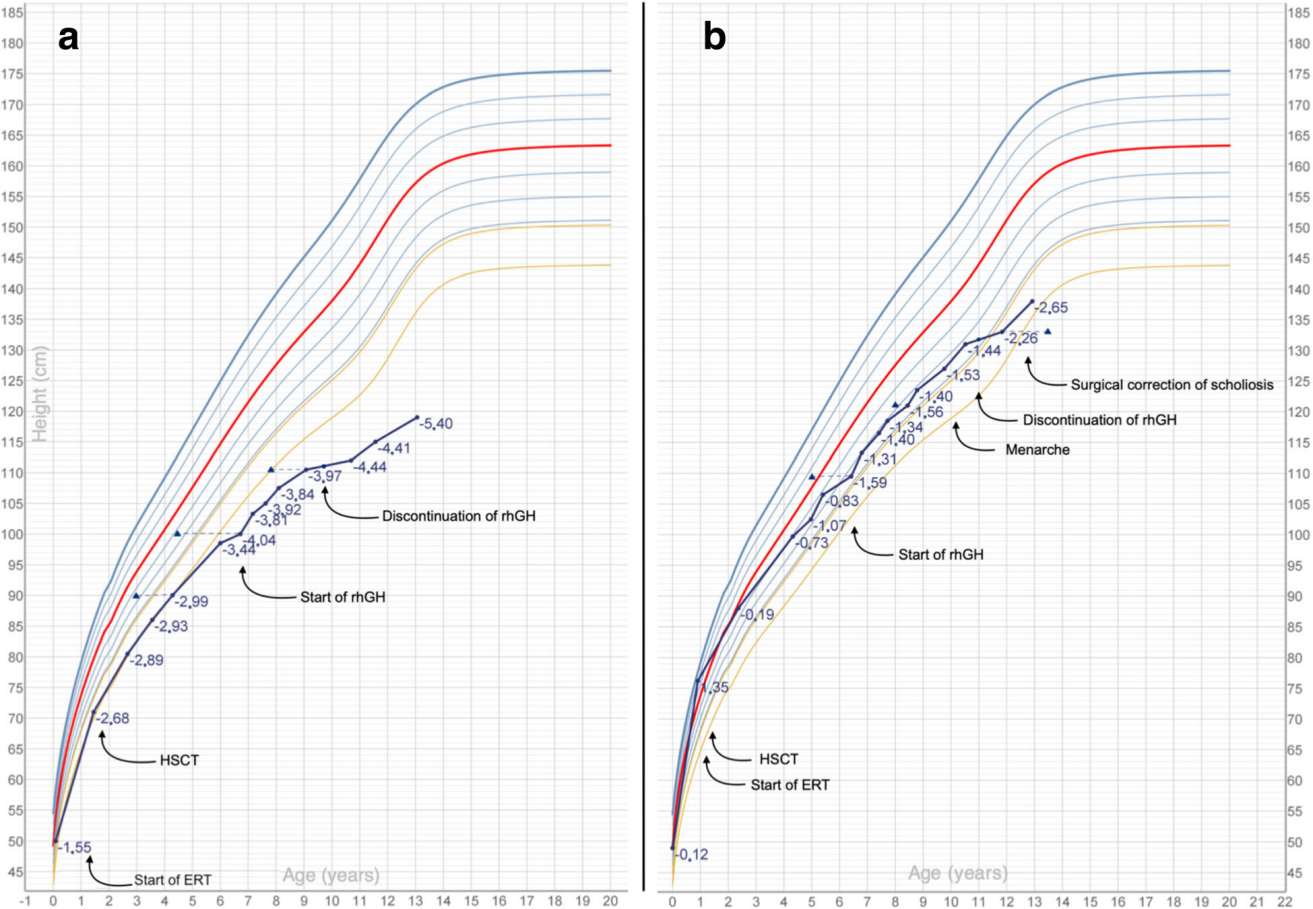
Legend: growth patterns for patients 1 (**a**) and 2 (**b**) – Blue dots represent height for age; height SDS for each measurement is expressed in blue; blue triangles represent height for bone age

Figure 1a shows patient’s growth chart before and after starting rhGH. The remarkable increase in height velocity recorded at 6 months (4.3 cm/year, − 2.11 SDS) and 12 months (5.2 cm/year, − 0.60 SDS) follow-up led to a mild improvement in height SDS (from − 3.57 to − 3.42 SDS after 12 months of treatment). While during the first 1.5 years of treatment the above-mentioned decline in height SDS was interrupted by the start of rhGH administration, a further progressive drop in height SDS was noticed subsequently and led the clinician to discontinue recombinant GH treatment at the age of 9.6 years given the overall poor late response (total duration of treatment: 2.9 years). The patient was pre-pubertal (Tanner’s stage: B1P1A1) before being started on treatment and the first early signs of pubertal development were noticed after the discontinuation of rhGH.

From an orthopaedic point of view, the skeletal abnormalities firstly found in early infancy showed a progressive worsening all throughout childhood.

A bilateral (right>left) coxofemoral sub-luxation was noticed 2 years before starting rhGH. A leg length discrepancy of about 1 cm was noticed about 2 years after the start and 1 year before the discontinuation of treatment. At last orthopaedic follow-up (3 years after the discontinuation of rhGH), the discrepancy had increased to 2 cm.

Also bilateral *genu valgum* showed a gradual worsening, but most of the progression occurred after the discontinuation of rhGH: a pre-treatment tibiofemoral angle of − 8° decreased to − 10° 6 months after the end of treatment and to − 12/− 15° at last orthopaedic follow-up, almost 3 years later.

Multiple vertebral malformations and T12-L1 spondylolisthesis had already been described in this patient since the age of 4 years.

Radiological follow-up showed further worsening of sagittal vertebral alignment about 1.5 years after rhGH discontinuation. Six months later (2 years after rhGH discontinuation), the patient started to complain about sporadic episodes of back-pain and lower limbs hyposthenia; hyporeflexia and progression of kyphosis were described at physical examination. The onset of clinical pyramidal signs raised the strict indication of a surgical treatment, at the age of 12.1 years.

In patient 2, ERT was started at the age of 13 months, while HSCT was performed 3 months later. The patient was diagnosed with GH deficiency when she was 6.4 years (GH peak at arginine test: 6.0 microg/L; GH peak at insulin tolerance test: 7.8 microg/L). Patient’s bone age, assessed at diagnosis of GH deficiency, was slightly delayed (5.0 years compared to a chronological age of 6.4 years). No abnormal findings involving the hypothalamic-pituitary area were noticed at MRI.

The start of rhGH was followed by a remarkable increase in height velocity: from 2.96 cm/year (− 3.44 SDS) at time 0 to 9.97 cm/year (+ 4.56 SDS) in 6 months’ time and 6.99 cm/year (+ 1.21 SDS) at the end of the first year of treatment. The initial decrease in height SDS that the patient had experienced between 5 and 6 years was interrupted by the start of recombinant GH and the patient’s growth kept on tracking the 10th centile until the age of 10.1 years, when she achieved menarche. Therapy with rhGH was discontinued when she was 11.0 years, as annual height velocity had dropped below 2 cm/year. IGF-1 levels rose from a baseline level of − 1.7 SDS to + 1.3–1.7 SDS during treatment.

Figure 1b shows the growth pattern reported for this patient. The steep increase in height showed after rhGH discontinuation is the consequence of the spinal surgical intervention she underwent at the end of growth.

From an orthopaedic point of view, a double kyphotic cervical curvature and lumbar scoliosis were noticed well before the rhGH treatment. Annual follow-up was performed during and after discontinuing rhGH and no worsening of spinal malformation was reported. As previously stated, the patient successfully underwent elective surgical correction of spinal kypho-scoliosis at the end of growth.

During the second year of life, hip X-rays showed initial coxo-femoral sub-luxation. Radiological follow-up highlighted a progressive worsening of these malformations before starting rhGH, with a more remarkable involvement of right femoral head and subsequent increasing leg length discrepancy. At the end of growth, right femoral head subluxation was radiologically more pronounced, but never progressed into overt luxation.

### Morquio syndrome (MPS IV)

A single patient with Morquio syndrome has been found to be GH-deficient at our centre (patient 3).

**Fig. 2:**
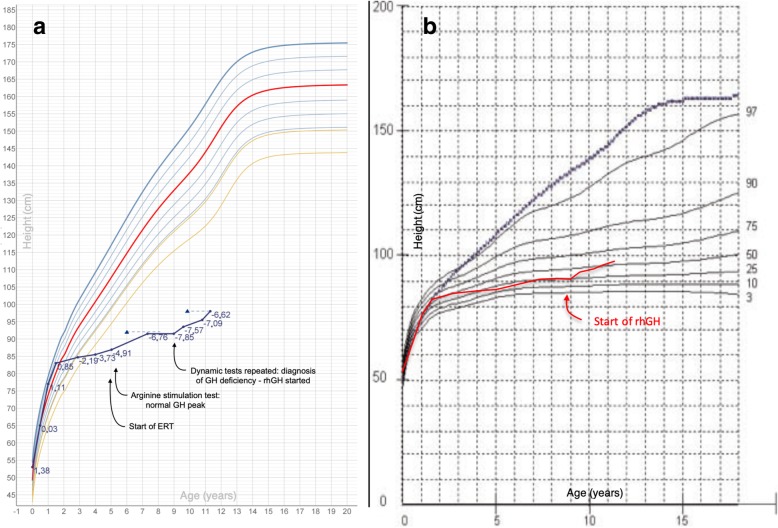
Legend: growth patterns for patient 3 plotted on CDC (**a**) and disease-specific growth charts (**b**, modified from reference [14]). In **a**, blue dots represent height for age; height SDS for each measurement is expressed in blue; blue triangles represent height for bone age. In **b**, the dotted line shows the 50th centile for height in non-affected girls

As shown in Fig. 2, after an early satisfactory growth during the first year of life, the patient experienced a significant decrease in height velocity. She underwent arginine stimulation test at the age of 5.3 years, which provided a normal peak. After a complete arrest of growth between 7.3 and 8.9 years, the patient was re-tested and GH deficiency was confirmed (arginine test: GH peak 5.7 microg/l; dexamethasone test: GH peak 6.1 microg/L). The radiological finding of a remarkably delayed bone age (6 years, compared to a chronological age of 8.3 years) was consistent with the diagnosis of GH deficiency.

Baseline IGF-1 SDS before starting rhGH was − 2.1 SDS. MRI highlighted a dysmorphic appearance of the sella and the sphenoid bone, as consistent with MPS morphology, with normal anatomy and volume of both adeno- and neurohypophysis.

After starting her on rhGH (initial dose: 0.027 mg/kg/die), height velocity showed a remarkable increase, from 0.16 cm/year (− 6.12 SDS) to 3.33 cm/year (− 1.33 SDS) at last clinical follow-up, 24 months later. These auxological findings were consistent with a normalization of IGF-1 SDS during treatment (+ 1.0 to + 1.5 SDS).

The progressive decrease in height SDS experienced between 4.1 and 8.9 years was therefore reverted by hormonal replacement treatment (Fig. 2a and b).

From an orthopaedic point of view, the patient did not experience any complication after 24 months of treatment with rhGH. *Genu valgum* was surgically corrected before starting the patient on hormonal treatment and no changes were described at subsequent radiological follow-up; *coxa valga* was diagnosed before starting rhGH and no further worsening described later on.

In addition, the repetition of a dual-energy X-ray absorptiometry (DXA) scan 1 and 2 years after starting rhGH showed a progressive improvement of bone mineral density: total body bone mineral density z-score raised from − 4.9 observed 1 month before starting rhGH to − 3.7 (1.6 years later) and − 2.3 (2.4 years later).

### Maroteaux-Lamy syndrome (MPS VI)

One female patient was started on rhGH due to demonstrated GH deficiency in our Centre (patient 4). The child had grown regularly during the first years of life (tracking the 50th centile for general population - CDC growth charts). After the age of 6 years, her height SDS began to show a progressive decrease. At the age of 8.8 years, she therefore underwent GH stimulation tests, which confirmed the diagnosis of GH deficiency (arginine test: GH peak 3.7 microg/L, dexamethasone test: GH peak 7.4 microg/L). Hand and wrist X-ray, performed at diagnosis of GH deficiency, showed a bone age of 7.8 years. Neither sellar nor juxtasellar abnormalities were noticed at MRI.

**Fig. 3:**
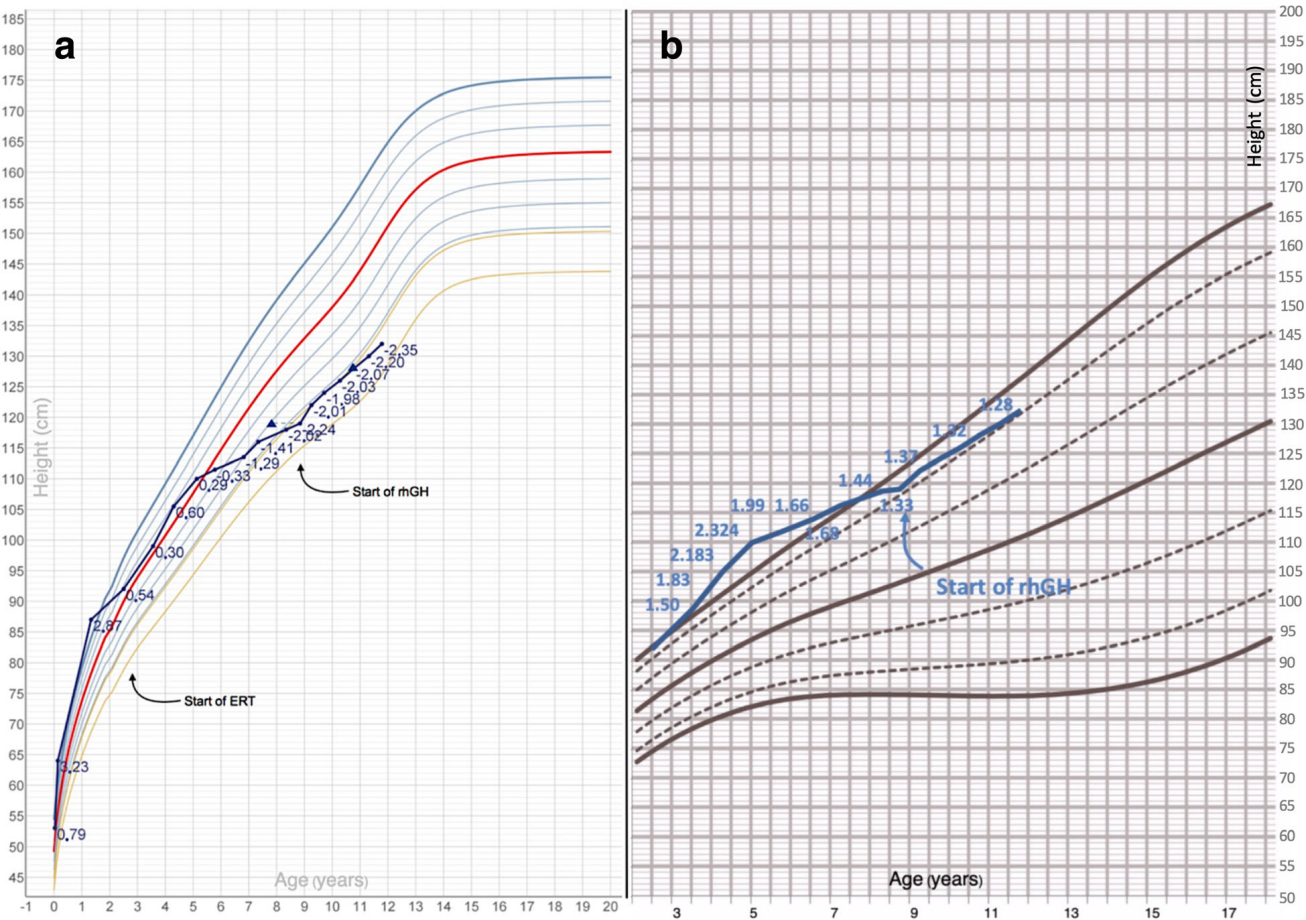
Legend: growth patterns for patient 4 plotted on CDC (**a**) and disease-specific growth charts (**b**, modified from reference [15]). In **a**, blue dots represent height for age; height SDS for each measurement is expressed in blue; blue triangles represent height for bone age

Treatment with rhGH was started at the age of 8.8 years. After the start of treatment, height velocity showed a remarkable increase (from − 4.32 SDS to − 0.95 after 6 months and + 0.95 at the end of the first year of treatment). This response reverted the previous trend towards a progressive decrease in height SDS and was therefore followed by a regular growth (height stably around + 1.3 SDS for disease-specific charts between 8.8 and 11.75 years, at last clinical follow-up) (Fig. 3a and b). IGF-1 SDS values fell within the normal range before (+ 1.1 SDS) and after (+ 1.4 to 1.9 SDS) starting rhGH.

From an orthopaedic point of view, a dorso-lumbar scoliosis diagnosed during the first years of life was treated conservatively with a back brace. In addition, *genu valgum* diagnosed before starting growth hormone replacement therapy was treated surgically with hemiepiphysiodesis, with a positive short- and long-term outcome; the overall orthopaedic picture showed no signs of progression after 3.8 years of treatment with rhGH.

Finally, the DXA scans performed before starting rhGH and 2.1 years later showed a remarkable increase in bone mineral density (total body bone mineral density z-score from − 1.4 to − 0.5).

## Discussion

In the present case series, we provide detailed anthropometric and orthopaedic figures about four MPS patients treated with rhGH due to a demonstrated GHD.

Some Authors have already analysed the effects of rhGH in patients affected by Mucopolysaccharidoses, but most data are drawn from cohorts of mostly non-GH deficient patients [3, 12, 13, 18]. As in most Countries therapy with recombinant growth hormone is licensed only for patients with a proven deficiency in somatotropin secretion and since the response to rhGH in non-GH deficient and GH-deficient patients is expected to be different, we focused on the latter class of patients.

The clinical *criterium* raising the suspicion of GHD and leading the clinicians to perform GH dynamic tests was growth deceleration or complete growth arrest for all the presented patients.

A remarkable increase in height velocity SDS at 6 and 12-months’ follow-up after starting rhGH (average Δ height velocity SDS: + 4.23 ± 2.9 at 6 months’ follow-up, + 4.55 ± 0.96 at 12 months’ follow-up) reverted the previous trend towards a progressive decrease in height SDS for all the patients. Like in most otherwise healthy patients treated with rhGH, height-velocity SDS started to show an initial decrease after 2 years of therapy (Δ height velocity SDS: + 3.2 ± 1.12). In one patient diagnosed with MPS IH and treated with HSCT, the initial response to rhGH was followed by a progressive decrease in height velocity and treatment was therefore discontinued after 2.9 years of therapy.

Our experience and the literature support the possible efficacy of rhGH in increasing height-velocity and reverting a progressive drop in height SDS in MPS patients with proven GH deficiency, at least for the first 2 years of treatment.

In a cohort of 23 patients with Hurler and Hunter disease, Polgreen and colleagues showed a non-statistically significant difference in height velocity between treated and non-treated subjects [12]. Conversely, our two MPS IH patients showed a remarkable increase in height velocity during the first year of treatment (from 1.8 to 5.32 cm/year and from 2.96 to 6.99 cm/year, respectively). This apparent discrepancy might probably be attributed to the fact that the study population in the cited paper mostly included patients with a normal somatotropin secretion, as only 4 children had a proven GH deficiency.

Both the MPS IH patients enrolled had undergone HSCT after a busulphan/cyclophosphamide conditioning. While the detrimental role of TBI on growth has been widely described, it has been historically assumed that busulphan−/cyclophosphamide-based regimens do not affect the final height attained after HSCT [19, 20]. Only more recently, Bakker and colleagues have presented data consistent with a possible degree of impaired GH secretion in patients treated with busulphan-based regimens, but very few data about the effect of rhGH in this cohort of patients are available [21]. As a consequence, the role of the cited conditioning scheme on growth deceleration in patients 1 and 2 and on the poor response to rhGH recorded in patient 1 can neither be confirmed nor excluded.

MPS I patients are known to have an early decline of growth and to decrease to the third centile around 30 months of life [22].

Montano and colleagues have described a progressive growth failure in 312 children with Morquio disease [14]. The mean adult height was 122.5 ± 22.5 cm for men and 116.5 ± 20.5 cm for women. In patients with MPS IV, despite a normal length in early childhood, height drops below − 2 SDS at an average age of 4 years, remarkably earlier than in children with MPS IH and MPS II [12].

Published reports suggest that MPS VI infants and toddlers have normal to accelerated growth velocity during the first year of life, followed by decreased growth rates during the second year [23]. The median height after the age of 3 years drops below the 5th centile for height of the age-adjusted peers in the normal population according to the CDC growth charts [15].

No data about GH-deficient patients with MPS IV or MPS VI are available in the literature. Polgreen and colleagues described anthropometric data about the use of rhGH in a patient with Morquio disease and one with Maroteaux-Lamy syndrome, but, again, they had normal response to GH stimulation tests [3]. To the best of our knowledge, we describe here the first MPS IV and MPS VI patients treated with rhGH because of proven GHD; our data demonstrate an early response of both height SDS and height velocity SDS.

Given the widely described detrimental effects of the pathologic accumulation of GAG in the bones and growth plates, the early effectiveness of rhGH in improving height velocity was not an obvious result.

The prevalence of GH deficiency among the population of MPS patients followed at our institution was remarkably higher than in the general paediatric population (1:4000–1:8000 children) [24, 25]. We speculate that this might be related both to the detrimental effect of HSCT on GH secretion and to GAG deposition in the pituitary [21].

The diagnosis of GH deficiency requires a combination of auxological (height SDS, height velocity SDS), clinical and biochemical (GH peaks achieved after dynamic tests) data. As growth deceleration can be at least partially attributed to mucopolysaccharidosis itself, one may argue that our patients (diagnosed with mild GHD and with normal radiological findings at MRI) could not be unequivocally labelled as GH-deficient. Actually, all the patients underwent GH stimulation tests because of complete growth arrest or severe growth deceleration, not expected even when compared with the MPS population. We therefore believe that both auxological and biochemical criteria for diagnosing GH deficiency were fulfilled.

At diagnosis of GH deficiency, bone age was remarkably delayed in comparison to chronological age for all the children enrolled, as expected in GH-deficient patients. The subsequent radiological follow-up, as reported in Figs. 1, 2 and 3, showed a progressive reduction of the gap between chronological age and bone age, also in prepubertal children. Bone maturation is a widely described effect of rhGH in otherwise healthy children diagnosed with GHD, but it is well known that it does not affect growth potential in the general paediatric population [26], at least in pre-pubertal GH-deficient patients treated with rhGH doses not greater than 0.035 mg/kg daily. In MPS patients, the effect of rhGH on final height and the role of rhGH-induced bone maturation have not been specifically assessed yet. Further analyses are required; however, the well described carpal bone dysplasia reported in patients with MPS [27] might result in a misleading assessment of bone age in these individuals.

Concerns about the use of rhGH in patients with multiple skeletal abnormalities have been raised because an increase in height velocity has been reported as a major risk factor for the onset of slipped capital femoral epiphysis (SCFE) and for the progression of scoliosis and kyphosis in this group of patients [18].

Conflicting data have been reported about the potential detrimental effects of rhGH on scoliosis in non-syndromic patients, with most outcomes supporting its safety [28, 29]. Among patients with underlying conditions related to higher prevalence rates of scoliosis, de Lind van Wijngaarden and colleagues have demonstrated in a randomized trial that Prader-Willi patients treated with rhGH do not experience a significant worsening of spinal curvatures if compared to non-treated subjects [30]; however, it is possible that the complexity and severity of skeletal abnormalities in patients with MPS does not allow to systematically extend the same reassuring conclusions among these patients.

From an orthopaedic perspective, given the natural history of the disease and the fact that the progression of possible orthopaedic complications only partially concurred with the treatment with rhGH, no clear conclusions about the skeletal effects of GH in this patient can be drawn.

In none of the patients described, overt SCFE had occurred at last clinical follow-up.

In addition, all the orthopaedic abnormalities showing a progression during or after replacement treatment had already been diagnosed before starting rhGH. One patient experienced a progressive radiological and clinical worsening of kyphosis after being started on rhGH, which led to orthopaedic corrective surgery.

In two out of four patients for whom DXA scans were available before and after starting treatment, bone mineral density showed a remarkable improvement: these results are in keeping with the positive effects of growth hormone on bone mineralization already described in literature [31].

We are aware of the potential limitations of our analysis: its retrospective nature and the small sample size may affect the significance of the conclusions drawn. In particular, the potential detrimental effects of rhGH cannot be assessed on a small case series. However, as we analysed the effects of rhGH in a selected cohort of patients diagnosed with a co-occurrence of two rare conditions (namely GHD and MPS), we believe that the information provided may rise interesting discussions in the scientific community.

## Conclusions

In conclusion, our case series highlights that the start of rhGH in GH-deficient patients with MPS was followed by a remarkable increase in height velocity for at least 12–24 months. Irrespectively of the specific type of MPS, whenever GH deficiency was the proven cause of an otherwise unexplained drop in height SDS over time, rhGH was a useful tool in reverting a progressive height decrease. In our patients, a correlation between the progression of orthopaedic abnormalities and rhGH treatment can be neither demonstrated nor excluded and further data are needed to assess any eventual correlation; however, rhGH seems to show a positive effect on bone mineralization also in MPS patients.

In consideration of the proven early benefits of rhGH, we recommend starting rhGH in MPS patients diagnosed with GHD. However, larger studies are needed to assess the impact of rhGH on final height in MPS patients.

## Data Availability

All the data generated or analysed during this study are included in the published article.

## Abbreviations

DEXA: Dual-energy X-ray absorptiometry
ERT: Enzyme replacement therapy
GAG: Glycosaminoglycans
GH: Growth hormone
GHD: Growth hormone deficiency
HSCT: Haematopoietic stem cell transplantation
ITT: Insulin tolerance test
MPS: Mucopolysaccharidoses
rhGH: Recombinant human growth hormone
SCFE: Slipped capital femoral epiphysis
SDS: Standard deviation score
TBI: Total body irradiation
